# Cold Exposure Induces Swine Brown Adipocytes to Display an Island-like Distribution with Atypical Characteristics

**DOI:** 10.3390/ijms26209871

**Published:** 2025-10-10

**Authors:** Zhenhua Guo, Lei Lv, Hong Ma, Liang Wang, Bo Fu, Fang Wang, Shuo Yang, Di Liu, Dongjie Zhang

**Affiliations:** 1 Key Laboratory of Combining Farming and Animal Husbandry, Institute of Animal Husbandry, Heilongjiang Academy of Agricultural Sciences, Ministry of Agriculture and Rural Affairs, No. 368 Xuefu Road, Harbin 150086, China; 2 Wood Science Research Institute of Heilongjiang Academy of Forestry, No. 134 Haping Road, Harbin 150080, China

**Keywords:** browning, fat, pig, *UCP3*

## Abstract

The original purpose of this study was to compare human and pig scRNA-seq data to determine why pigs do not have brown adipocytes. However, during the experiment, we identified brown adipocytes in pigs. Therefore, we aimed to confirm that these adipocytes were brown adipocytes via a comparative analysis using typical mouse brown adipose tissue sections. We found that swine brown adipocytes were distributed in an island-like pattern, with three typical characteristics: (1) numerous mitochondria and small lipid droplets, (2) a cellular volume smaller than that of white adipocytes, and (3) expression of specific marker genes (*EBF2* and *ATP2B4*). The expression levels of the thermogenesis-related genes *UCP2*/*3* were not significantly increased. Thus, we conducted ceRNA network analysis, revealing that high expression of the key microRNA miR-10383 increased the thermogenic efficiency of *UCP3* in the cold exposure group. In addition, the epigenetic memory of *UCP3* was disrupted. Chromatin accessibility and Whole-Transcriptome Sequencing of Groin Adiposesibility results revealed peaks in the promoter regions of the *UCP2*/*3* genes. In our discussion of the study’s limitations, we explain how to repeat the experiment to significantly increase the UCP2/3 protein content. This study fills a research gap regarding brown fat in pigs and can provide a reference for future studies on fat metabolism.

## 1. Introduction

White and brown adipocytes in adipose tissue jointly regulate energy deposition [1]. Both humans and mice possess brown adipose tissue [2,3,4,5]. Brown adipose tissue apparently evolved to generate heat in response to cold [6,7,8,9,10]. Recent studies have revealed that the function of brown adipocytes extends beyond the suppression of obesity and associated metabolic diseases [11,12,13]. Brown adipocytes induced by cold exposure may also possess cancer-inhibiting properties [6], and adipose manipulation and transplantation have shown potential in cancer therapy [14]. Cold exposure is a classic method for inducing the white-to-brown transformation of adipocytes and is considered a potentially safe and effective treatment for obesity [6,7,15]. Thermogenic adipocytes include beige adipocytes (brite adipocytes) and brown adipocytes [16,17,18,19]. Despite reports of the discovery of beige adipocytes in pigs [20,21,22,23], there have been no reports of brown adipocytes in these animals to date. Brown adipocytes demonstrate the following three characteristics: (1) numerous mitochondria and small lipid droplets [19,24], (2) a smaller cellular volume than that of white adipocytes due to the consumption of intracellular lipid droplets for thermogenesis, and (3) the expression of specific marker genes (*EBF2* and *ATP2B4*).

Mitochondria are involved in thermogenesis via H^+^ leakage (*I*_H_) across the inner membrane. This thermogenic process is achieved by uncoupling the flow of H^+^ ions between the electron-transport chain and ATP synthase. In humans and mice, brown adipocytes execute H^+^ transport through uncoupling protein 1 (*UCP1*) [6,8,9,25,26]. However, the *UCP1* gene in pigs is defective, and this function may occur via uncoupling protein 2/3 (*UCP2*/*3*) [27,28,29,30]. Single-cell RNA sequencing can be used to precisely distinguish cell types and subclasses according to the expression of marker genes [31,32], and many reports on adipocyte marker genes have been published. White adipocytes specifically express *FABP5* [7], *LPIN1* [4], *GLUL* [4], and *PDGFRA* [33], and brown adipocytes specifically express *EBF2* [7], *ATP2B4* [7], and *COBL* [5,34]. Beige adipocytes specifically express *CD137* [20] and *ATP5K* [35].

The original purpose of this study was to determine why pigs do not have brown adipose tissue. However, during the experiment, we observed brown adipose tissue in these animals. Thus, we conducted further analyses to confirm that the cells we identified were indeed brown adipocytes.

## 2. Results

### 2.1. Human Brown Adipocyte scRNA-Seq Data Analysis

**Figure 1:**
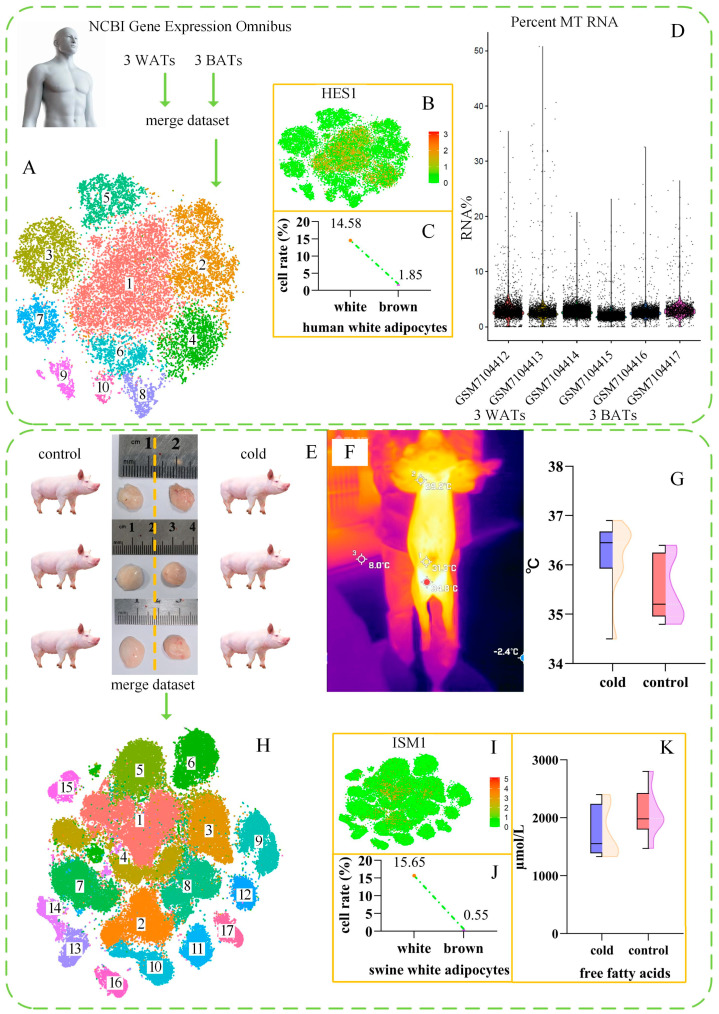
Comparative Analysis of Human and Swine Brown Adipocytes. (**A**) TSNE clustering analysis of scRNA-seq data from six human adipose tissues, resulting in 10 clusters, with cluster 1 identified as white adipocytes and cluster 3 identified as brown adipocytes. (**B**) The distribution of the *HES1* gene across various clusters. (**C**) White adipocytes are 12.73% more abundant in white adipose tissue than in brown adipose tissue in humans. (**D**) The percentages of MT RNA in human white and brown adipose tissues. (**E**) Compared to those of the control pigs, the inguinal fat of the cold-exposed pigs appears browner. (**F**) Surface and inguinal temperatures of pigs. (**G**) Comparison of inguinal temperatures between the cold exposure and control groups of pigs, with no significant differences observed. (**H**) TSNE clustering analysis of scRNA-seq data from six swine adipose tissues, resulting in 17 clusters, with clusters 1 and 3 identified as white adipocytes and clusters 2 and 4 as brown adipocytes. (**I**) The distribution of the *ISM1* gene across various clusters. (**J**) White adipocytes are 15.1% more abundant in white adipose tissue than in brown adipose tissue in pigs. (**K**) Comparison of free fatty acid concentrations in the blood of cold-exposed and control pigs by ELISA, with no significant differences observed.

Single-cell sequencing data from human white and brown adipose tissues (GSE227635) were downloaded from the GEO database, and the data from six adipose tissue samples were analyzed. The results for 10 clusters are shown in Figure 1A. According to existing human adipose tissue data, white adipocytes specifically express *FABP5*, whereas brown adipocytes specifically express *EBF2* [7]. In this study, cluster 1 was identified as being composed of white adipocytes, and cluster 3 was identified as comprising brown adipocytes. The expression levels of marker genes in the clusters in humans identified in the analysis are listed in Supplementary Table S7. Figure 1B and Supplementary Figure S1A show several white adipocyte marker genes; clusters 1 to 4, which are considered to represent different stages of adipocyte transformation, demonstrate a high degree of correlation. Figure 1C shows the percentage of human white adipocytes in white and brown adipose tissue, indicating a difference of 12.73%. Given that white adipocytes are the dominant cell type in adipose tissue, we concluded that cluster 1 represented white adipocytes alone. In addition, brown adipocytes have a high mitochondrial content [19,24], and the analysis confirmed the MT RNA percentage (Figure 1D); however, no significant difference was observed in the MT RNA percentage between white and brown adipose tissue.

### 2.2. Swine Brown Adipocyte scRNA-Seq Data Analysis

The inguinal fat of pigs exposed to cold temperatures appeared to contain brown adipocytes, in contrast to the control animals (Figure 1E). However, there was no significant difference in surface body temperature between the two groups of pigs (Figure 1F,G). An analysis of six pig adipose tissue sample datasets revealed 17 clusters (Figure 1H). Given the specific expression of markers of white adipocytes, such as *CIDEA* [27,36], *LPIN1* [4], and *GLUL* [4], and brown adipocytes, such as *EBF2* [7] and *ATP2B4* [7], we identified clusters 1 and 3 as white adipocytes and clusters 2 and 4 as brown adipocytes. The identified marker genes of clusters in swine are listed in Supplementary Table S8. Figure 1I and Supplementary Figure S1B show several marker genes (*ISM1*, *ANGPTL1*, *AEBP1*) of white adipocytes, revealing strong correlations among clusters 1 to 4. Figure 1J shows the percentages of white adipocytes in white and brown adipose tissue in pigs, with a difference of 15.1%. We identified brown adipocyte clusters in both swine and human white adipose tissues.

### 2.3. Histological Evidence of Swine Brown Adipocytes

**Figure 2:**
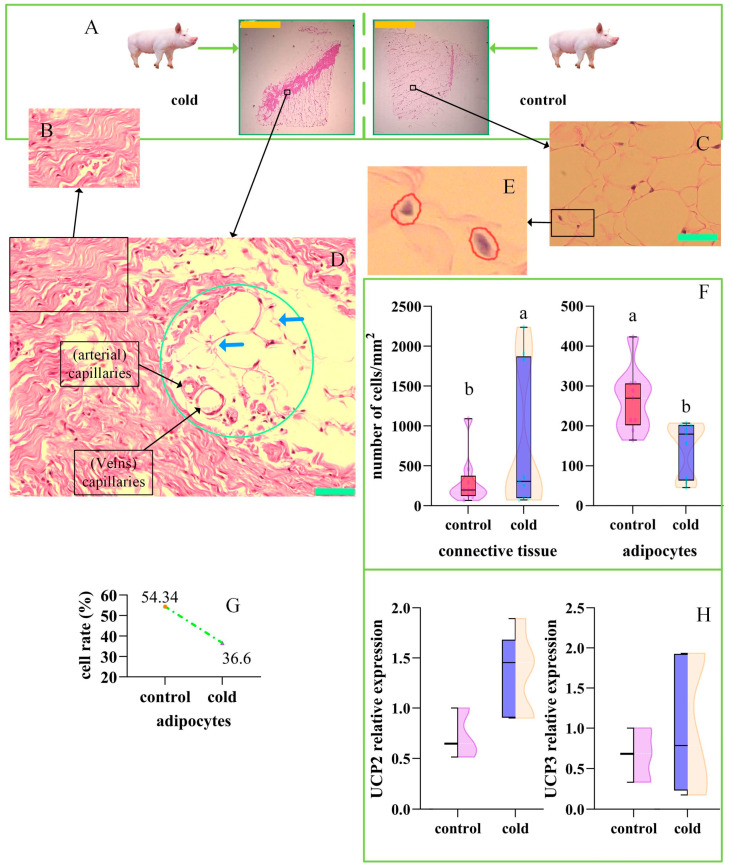
Cold Exposure Induces Morphological Changes in the Inguinal Adipose Tissue of Pigs. (**A**) Cold exposure induced morphological changes in the inguinal adipose tissue of pigs, with the brown bar representing 3 mm. (**B**) Typical connective tissue. (**C**) Typical adipose tissue, with the green bar representing 50 µM. (**D**) The green circle indicates the island-like distribution of brown adipose tissue. The overall layout is finger-like, with a local island-like distribution. The blue arrows indicate small lipid droplets. (**E**) Nuclei captured by QuPath. (**F**) The number of adipocytes and connective tissue cells in the cold-exposed and control groups. The difference in connective tissue cell count was significant (*p* = 0.0072, *t*-test), as was the difference in adipocyte count (*p* = 0.0037). (**G**) Compared to the control group, the cold exposure group had 17.74% fewer adipocytes. (**H**) Significance analysis of *UCP2*/*3* genes in adipose tissue, showing no significant differences between the cold exposure and control groups.

Compared to the control group, the inguinal adipose tissue of pigs in the cold exposure group demonstrated increased connective tissue (Figure 2A). Figure 2B,C illustrate typical connective tissue and adipocytes, respectively. Notably, adipocytes with an island-like distribution were found at the adipocyte–connective tissue junction (Figure 2D). These cells were smaller in volume than the other adipocytes and were surrounded by blood vessels. Thus, we identified these cells as swine brown adipocytes and conducted further evaluation for confirmation. The cells were counted using a nuclear counting approach (Figure 2E), revealing a significant increase in the number of connective tissue cells in the cold exposure group, whereas the number of adipocytes was significantly decreased (Figure 2F). The number of adipocytes in the pigs was decreased by 17.74% after cold exposure (Figure 2G).

**Figure 3:**
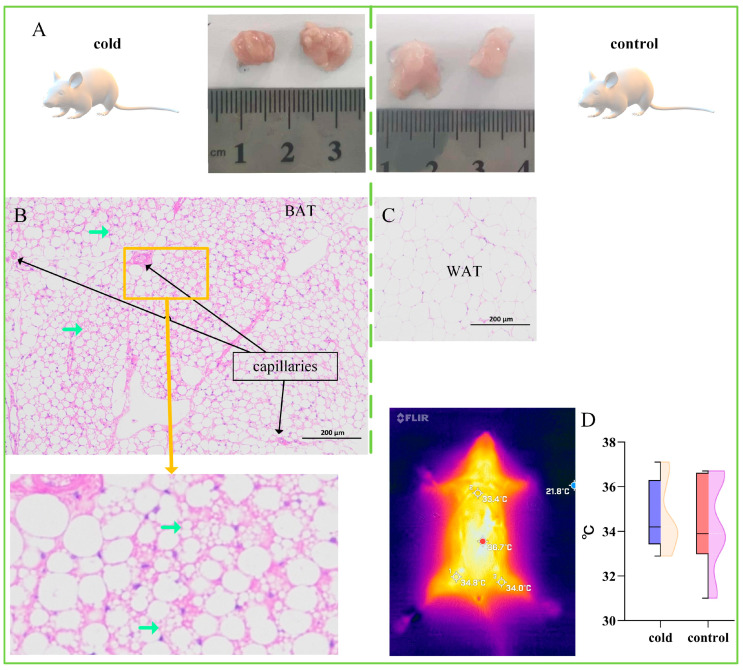
Cold Exposure Induces the Interscapular Fat of Mice to Exhibit Typical Brown Adipocytes. (**A**) The interscapular fat of the mice in the cold exposure and control groups showed a brownish color change. (**B**) The interscapular fat of the mice in the cold exposure group exhibited typical brown adipose tissue, with a relatively small adipocyte volume. Numerous multilocular lipid droplets in adipocytes can be observed, with the green arrows indicating multilocular lipid droplets in the adipocytes. (**C**) The interscapular fat of the mice in the control group exhibited typical white adipose tissue, with increased adipocyte volume. (**D**) Thermographic images of the surface temperature of mice and comparisons of the surface temperature between the cold exposure and control groups of mice. There was no significant difference between groups.

In mice, the cold exposure group exhibited a brownish color in the interscapular fat relative to the control group (Figure 3A), with typical brown adipocytes present in this region (Figure 3B). The control group presented typical white adipocytes (Figure 3C). There was no significant difference in surface body temperature between the two groups of mice (Figure 3D). These results suggested that the distribution of swine brown adipocytes differs from that in mice (Figure 2D), indicating atypical characteristics in pigs. Moreover, brown adipose tissue in both mice and humans contains more UCP1 protein and shows higher expression of *UCP2* than white adipose tissue. There was no significant difference in the expression levels of *UCP2*/*3* (Figure 2H) in the adipose tissue of the cold exposure group, representing another atypical characteristic.

### 2.4. Subcellular Structural Evidence of Swine Brown Adipocytes

**Figure 4:**
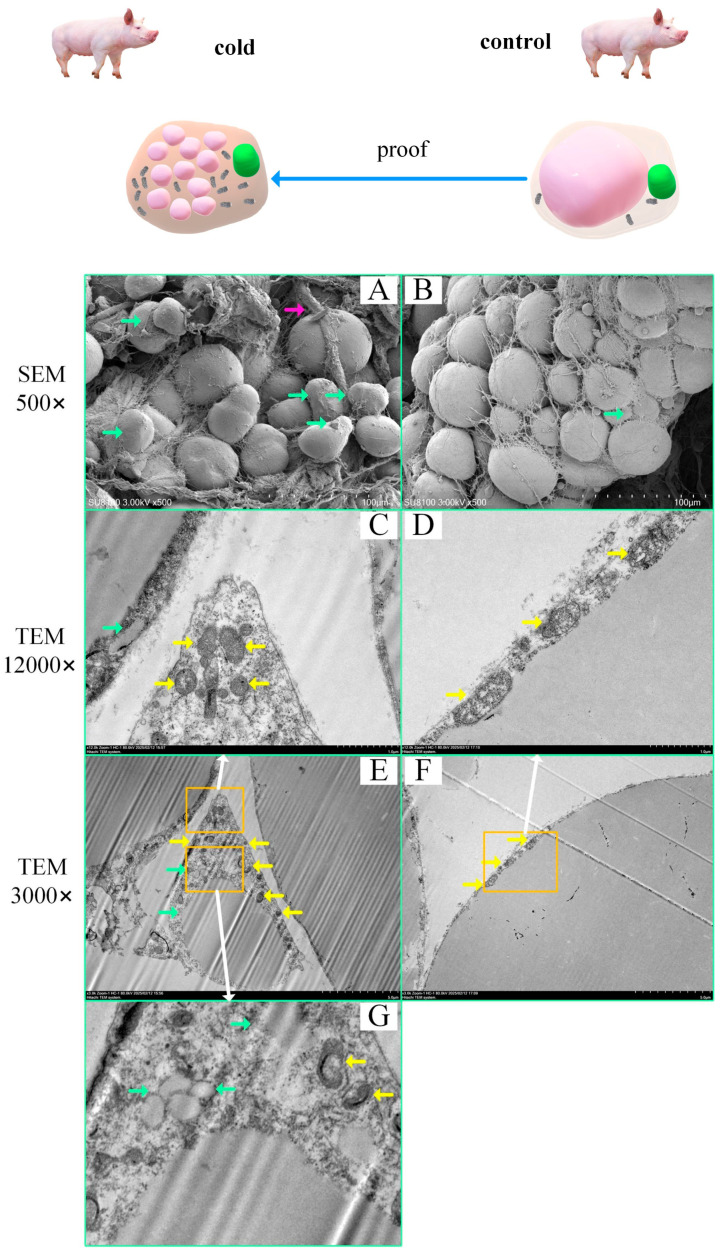
Cold Exposure Induces Subcellular Structural Changes in the Inguinal Adipose Tissue of Pigs. (**A**,**B**) Scanning electron microscopy images of the cold exposure and control groups, with the green arrows indicating multilocular lipid droplets in adipocytes and the purple arrows indicating blood vessels or lymphatic vessels. (**C**–**G**) Transmission electron microscopy images of the cold exposure and control groups. (**E**) A typical brown adipocyte showing multiple small lipid droplets within the cell and a greater number of mitochondria. (**C**,**G**) are close-ups of (**E**). (**D**) is a close-up of (**F**). The orange arrows indicate mitochondria.

Transmission and scanning electron microscopy images revealed brown adipocytes at the adipose–connective tissue junction in cold-exposed pigs. More multilocular lipid droplets were observed in the adipocytes of the cold exposure versus the control groups (Figure 4A,B). Transmission electron microscopy revealed a greater number of mitochondria in the cold exposure versus the control groups (Figure 4C–G). These characteristics are typical of brown adipocytes. Furthermore, we observed brown adipocytes with multilocular lipid droplets on the verge of complete consumption (Figure 4E). The cells contained multiple small lipid droplets (Figure 4G); increased energy consumption is believed to cause the integration of brown adipose cells into the connective tissue, making it difficult to distinguish the features of brown adipocytes. The previous scRNA-seq findings in swine and human white adipose tissues identified clusters of brown adipocytes, confirming the presence of brown adipocytes in the inguinal white adipose tissue of pigs (Figure 4B; the green arrows indicate multilocular lipid droplets).

### 2.5. Changes in the Serum Components of Pigs After Cold Exposure

Further experiments were conducted to elucidate the mechanism underlying the changes in pig adipose tissue following exposure to cold. Cold exposure did not alter the serum concentration of free fatty acids (FFAs) (Figure 1K). However, cold exposure significantly reduced the serum insulin content (Supplementary Figure S3) and significantly increased the levels of glutathione peroxidase, malondialdehyde, and creatine kinase (Supplementary Figure S3). The levels of triiodothyronine (T3), thyroxine (T4), superoxide dismutase (SOD), and cortisol were unaffected, as was the total antioxidant capacity.

### 2.6. Whole-Transcriptome Sequencing of Groin Adipose Tissue

**Figure 5:**
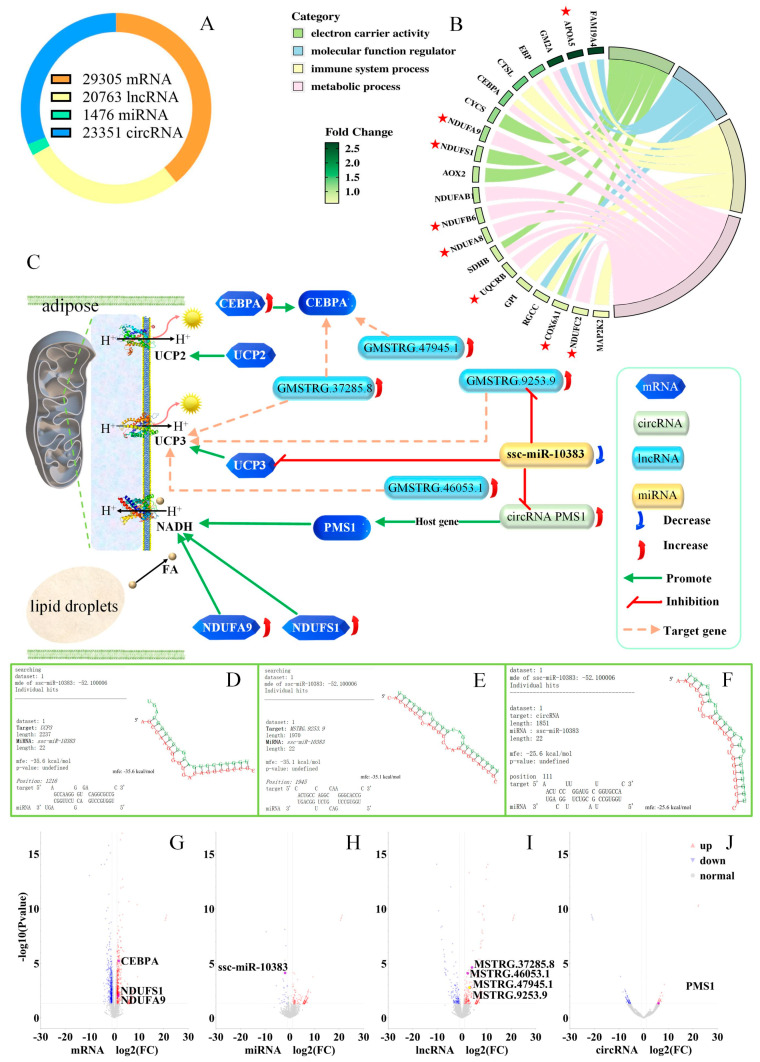
Whole-transcriptome Sequencing After Cold Exposure to Identify the ceRNA Network. (**A**) A total of 18 samples were analyzed, and mRNAs, lncRNAs, circRNAs, and miRNAs were detected. (**B**) GO enrichment analysis of upregulated genes in subcutaneous fat from the cold-exposed group. (**C**) ceRNA network in subcutaneous fat from the cold-exposed group, with FAs representing fatty acids in the figure. (**D**–**F**) Docking simulations of the miR-10383 miRNA with *UCP3* mRNA, GMSTRG.9253.9 lncRNA, and the PMS1 circRNA. (**G**–**J**) Differentially expressed mRNAs, miRNAs, lncRNAs, and circRNAs in subcutaneous fat from the cold-exposed group compared to the control group.

No significant changes in the *UCP2*/*3* protein or gene expression levels were observed after cold exposure. There are two possible reasons for this result. First, changes in expression may have occurred within the 63-h experimental process and returned to normal before the measurements were performed. Second, the competitive endogenous RNA network (ceRNA network) may have played a positive role in regulating *UCP2/3* expression in response to cold temperatures. To further investigate the underlying mechanism involved, mRNA, long non-coding RNAs (lncRNA), circular RNAs (circRNA), and miRNA analyses were conducted on the subcutaneous fat, liver, and longissimus dorsi muscle of three cold-exposed and three control pigs. A total of 29,305 mRNAs were detected in the 18 samples (Figure 5A). Circos plots were generated to determine the significance of differentially expressed RNAs in the subcutaneous fat, liver, and longissimus dorsi muscle (Supplementary Figure S4A, B, and C, respectively). These data served as clues for identifying the ceRNA network. The differentially expressed mRNAs, lncRNAs, circRNAs, and miRNAs between the cold exposure and control groups are shown in Venn diagrams (Supplementary Figure S4D (subcutaneous fat), S4E (liver), and S4F (longissimus dorsi muscle)). The mRNAs significantly upregulated in the cold exposure versus control groups were subjected to Kyoto Encyclopedia of Genes and Genomes (KEGG) enrichment analysis, which identified pathways related to oxidative phosphorylation, thermogenesis, and fatty acid metabolism (Supplementary Figure S5A–C). A Gene Ontology (GO) enrichment plot of the upregulated genes was generated (Figure 5B), with the starred genes participating in the oxidative phosphorylation pathway.

### 2.7. ceRNA Network

A ceRNA network analysis was conducted for the differentially expressed mRNAs, miRNAs, and circRNAs between the cold-exposed and control groups. Figure 5B highlights the key miRNA miR-10383, a crucial factor in the response to cold exposure. This miRNA can inhibit *UCP3* mRNA (Figure 5D), the lncRNA GMSTRG.9253.9 (Figure 5E), and the circRNA PMS1 (Figure 5F). We hypothesized that the lncRNA binds to the UCP3 protein, protecting it from enzymatic degradation, thereby enhancing the protein’s efficacy without altering its quantity. The differentially expressed mRNAs, miRNAs, lncRNAs, and circRNAs are, respectively, annotated in Figure 5G–J.

### 2.8. DNA Methylation Epigenetic Memory

**Figure 6:**
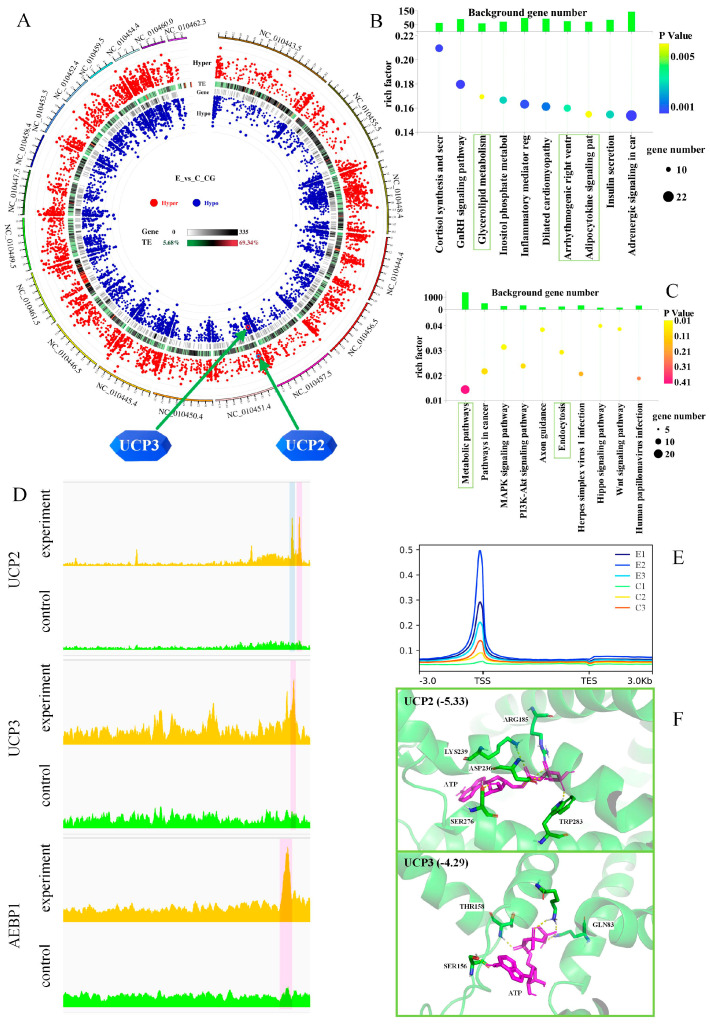
Changes in DNA Methylation After Cold Exposure and Subsequent Return to a Comfortable Temperature. (**A**) DMRs at CG sites in the experimental group compared to the control group according to WGBS. (**B**,**C**) KEGG enrichment analysis of hyper-down and hypo-up methylated DMRs. (**D**) The ATAC–seq distribution of normalized reads for the *UCP2*/*3* and *AEBP1* genes. The translucent pink and blue boxes in the figure represent peaks in the 3 kb region upstream and downstream of the TSS and within 1 kb of the TSS, respectively. (**E**) The distribution of reads relative to the body. (**F**) Molecular docking of UCP2 and UCP3. The yellow dashed line in the figure represents a connection bond of less than 5 Å.

We did not investigate the exact time within the 63-h experimental process at which *UCP2*/*3* expression significantly increased. Considering the epigenetic memory of adipose tissue, after 63 h of exposure, the pigs in the cold exposure group were transferred to the control temperature for an additional seven days. The epigenetic differences between the experimental (*n* = 3) and control (*n* = 3) groups were subsequently compared. The *UCP3* gene exhibited hypo-up methylation at CG sites, whereas the *UCP2* gene exhibited hyper-down methylation (Figure 6A). However, there were no differences at the CHH and CHG sites (Supplementary Figure S6A,B). The intersection of differentially methylated regions (DMRs) in the promoters and gene bodies at the CG, CHG, and CHH sites was assessed (Supplementary Figure S6C,D). The intersecting genes were merged and analyzed with the scRNA-seq marker genes of clusters in swine (Supplementary Table S9), revealing that the intronic CG site of the marker gene adipocyte enhancer-binding protein 1 (*AEBP1*) in cluster 1 was hypomethylated.

KEGG enrichment results for the hyper-downregulated pathways revealed the involvement of glycerolipid metabolism, the adipocytokine signaling pathway, and insulin secretion (Figure 6B). Conversely, the pathways associated with hypomethylation included metabolic pathways and endocytosis. These findings suggest that lipid droplets can overflow from adipose tissue and be taken up by other cells through endocytosis, consistent with the observed movement of lipid droplets in *Drosophila* kidneys [37].

### 2.9. Chromatin Accessibility of Epigenetic Memory

A transposase-accessible chromatin high-throughput sequencing (ATAC–Seq) assay was employed to detect differences in chromatin accessibility between the experimental (*n* = 3) and control (*n* = 3) groups. The distribution of reads across the genome (Supplementary Figure S6E) and the distribution of reads in the region 3 kb upstream and downstream of the transcription start site were determined (TSS; Supplementary Figure S6F). In addition, we assessed the distribution of reads relative to the gene body, with the experimental group showing higher read counts near the TSS than the control group (Figure 6E). The alignment of the *UCP2*, *UCP3*, and *AEBP1* genes (Figure 6D) revealed that, in the experimental group, all three genes presented a peak located 2–3 kb from the promoter. *UCP2* exhibited an additional peak < 1 kb from the promoter.

### 2.10. UCP2 and UCP3 Molecular Docking

The results of UCP2 and UCP3 protein docking were compared (Figure 6F), revealing that UCP2 (binding affinity of −5.33 kcal/mol) bound to ATP more strongly than UCP3 (binding affinity of −4.29 kcal/mol). Since hydrogen ions are passively transported out of the mitochondrial inner membrane through UCP2/3, the lower binding affinity of UCP3 suggests that it may be the primary transport pathway.

## 3. Discussion

In this study, we identified brown adipocytes in the inguinal adipose tissue of cold-exposed Large White pigs. These cells were confirmed to be brown adipocytes through histological and gene expression analyses. The brown adipocytes in pigs do not exist in large numbers, as they do in mice, and the temperature increase in such adipose tissue is insufficient to be reflected on the body surface (Figure 1G,H and Figure 3D).

Pigs are important animal models for human diseases [38,39,40], and potential donor animals for human organ transplantation [41,42,43]. Comparative anatomy and histology currently lack information concerning beige adipose tissue in humans and swine [44,45]; however, there are differences in local anatomy among animals. For example, the ovaries of humans and mice have an exterior cortex and an interior medulla, while the ovaries of horses are arranged in the opposite way [46]. Furthermore, humans and mice have discoidal placentas, whereas pigs have a diffuse placenta [47].

### 3.1. The Possibility of the Presence of Brown Adipocytes in White Adipose Tissue

Brown adipocytes are known to be distributed within white adipose tissue. Studies have shown that brown adipocytes are present in the thoracic cavity, head, neck, abdomen, inguinal region, and pelvis in mice [5,48,49]. NPY, which is found in the white adipose tissue of mice, can facilitate white-to-brown adipocyte conversion, thereby reducing body weight through thermogenesis [2]. The scRNA-seq results in this study also revealed the presence of brown adipocytes in white adipose tissue, which was confirmed by scanning electron microscopy (Figure 4B). Cold exposure stimulates adipose tissue to secrete batokines, which promote angiogenesis and the formation of brown adipocytes [50,51,52]. Furthermore, an increase in adipose connective tissue has been observed in mice after cold exposure [53]. Other studies have also shown that cells associated with blood vessels and immune cells play crucial roles in regulating the differentiation and function of brown and beige fat [54,55]. Human brown and beige adipocytes have different developmental origins and express different marker genes [5].

Consistent with the above reports, this study revealed that after cold exposure, the content of connective tissue in the inguinal fat of pigs increased, and brown adipocytes were distributed in an island-like pattern near the capillaries (Figure 2D and Figure 4A).

### 3.2. Histological Characteristics of Brown Adipocytes

In recent years, the discovery of brown adipocytes in adult humans has created the possibility of safer and more effective treatments for obesity and related metabolic diseases [5,12,15,56,57]. Previous studies have identified “brown-like” adipocytes in porcine adipose tissue [58], and the transmission and scanning electron microscopy images are very similar to those in our study. The most typical characteristic of brown adipocytes is the presence of multiple small lipid droplets within the cytoplasm [1,12], along with numerous mitochondria that work in concert with the lipids to produce heat [19,24]. In this study, we confirmed the presence of brown adipocyte characteristics in cold-exposed pigs (Figure 4E), indicating that our conclusions are supported by subcellular structural evidence.

In white adipose tissue, the combined proportion of white adipocytes and brown adipocytes accounts for 20–30% of the total cell count [13]. In this study, we demonstrated that the percentage of human white adipocytes differed between white and brown adipose tissue, with a difference of 12.73% (Figure 1C). In pigs, the difference in the percentage of white adipocytes between white adipose tissue and brown adipose tissue was 15.1% (Figure 1J). The HE staining of porcine white and brown adipose tissues revealed that the number of adipocytes in pigs decreased by 17.74% after cold exposure (Figure 2G). Adipocytes accounted for approximately 50% of the total cell count (Figure 2G), and summing the cell numbers of clusters 1 to 4 yielded a figure near this percentage. Thus, the clustering analysis results were supported by the histological findings.

### 3.3. Gene Expression Characteristics of Brown Adipocytes

Strict quality-control thresholds can exclude up to 50% of the nuclei in snRNA-seq, compared to ~10% of cells in scRNA-seq [5,59]. Therefore, we used the more accurate scRNA-seq approach. Independent scRNA-seq data constitute a flat matrix, and the analysis method is crucial [60]. We combined scRNA-seq data from six individuals to establish a three-dimensional matrix and then reanalyzed the data to distinguish white from brown adipocytes. Clusters were established for both humans and pigs (Figure 1A,H), and brown adipocytes were identified in pigs. Clusters 1–4 in humans and clusters 1–4 in pigs presented significant similarity (Figure 1B,I). However, identifying specific adipocyte subclasses was not the objective of this study. Clusters 1-4 may represent different stages of adipocyte transformation [3]. *UCP1* is a key marker gene for brown fat in humans and mice [16,61,62], but is defective in pigs [22]. Thus, we focused on the UCP2 and UCP3 proteins. No significant changes in UCP2/3 gene expression or protein levels were observed after cold exposure (Figure 2H). There are two possibilities for this result. First, changes in expression may have occurred within the 63-h experimental process and then returned to normal before the analysis was conducted [4,52,63]. Second, the ceRNA network may have played a positive role in regulating UCP2/3 expression in response to cold temperatures [64,65]. Notably, it is also possible that both of these situations occurred simultaneously.

Brown adipocytes are regulated at multiple levels, and ceRNA [65], histone modification [63,66,67], DNA methylation [63,68,69], and RNA methylation [65] all participate in this regulation, which is also related to chromatin accessibility [64,70]. The whole-transcriptome analysis employed in this study revealed that the significantly upregulated mRNAs in the cold exposure versus control groups were enriched in thermogenesis in all three tissues tested and in the oxidative phosphorylation pathway in the subcutaneous fat and liver samples (Supplementary Figure S5). These findings indicate that all three tissues underwent thermogenic activity above the basal metabolic level in response to cold exposure [6,15,20,26,29]. The oxidative phosphorylation pathway involves the consumption of NADH and active pumping of H^+^ into the mitochondrial inner membrane by fatty acids, followed by passive pumping of H^+^ out by UCP2/3, generating heat in the process (Figure 5C) [27]. Increased expression of NDUFA9, NDUFS1, and CEBPA indirectly promotes this process. NDUFA9 [71,72], and NDUFS1 [73,74] can all regulate NADH, consistent with our findings.

In this series of processes, miR-10383 directly inhibits *UCP3* mRNA, the lncRNA GMSTRG.9253.9, and the circRNA *PMS1*. The host gene of the circRNA *PMS1*, *PMS1*, forms the NADH–PMS system for H^+^ transfer in plants [75,76]; however, there are currently no reports concerning this process in animals. The downregulation of miR-10383 combined with the upregulation of *NDUFA9*, *NDUFS1*, and *CEBPA* mRNAs, as well as the increased expression of the lncRNA GMSTRG.9253.9 and the circRNA *PMS1*, collectively enhance the efficiency of UCP2/3 proteins, enabling the tissue to produce more heat to meet physiological demands (Figure 5C–J). This explains the lack of significant differences in the protein levels of UCP2/3 (Figure 2G,H). Notably, changes may have occurred during the 63-h experimental process and then returned to normal before the analysis; other studies have reported significant increases in the *UCP3* gene within 4–48 h [29,30,36].

The fatty acid metabolism pathway was enriched in the subcutaneous fat and longissimus dorsi muscle (Supplementary Figure S5). However, the concentration of FFAs in the blood remained unchanged (Figure 1K), indicating that the metabolized FFAs were directly converted into heat [77,78,79,80]. Compared to those in the control group, the serum malonaldehyde and creatine kinase levels were increased in the cold exposure group (Supplementary Figure S3), suggesting a possible association with shivering thermogenesis [81]. However, we did not observe shivering in the cold exposure group. Previous studies have shown that cold exposure leads to increased creatine kinase levels [82,83,84], consistent with our results. Moreover, a decrease in insulin promotes lipolysis [85,86].

### 3.4. Changes in DNA Methylation After Cold Exposure and Subsequent Return to Warm Temperatures

The goal of this study was to confirm that the adipocytes identified were brown adipocytes. Our epigenetic modification data only showed whether the expression of the *UCP2*/*3* genes was significantly increased during the 63-h cold exposure period. Changes in DNA methylation in adipose tissue after cold exposure are characterized by epigenetic memory [4,52,63]; thus, detecting DNA methylation within a certain period after cold exposure is indicative. We aimed to determine whether the cold-exposed group could respond rapidly to subsequent cold exposure after being returned to normal temperatures. Thus, day 10 was selected for sample collection. The *UCP3* gene exhibited hypomethylation (Figure 6A), a response that can prepare the animal for subsequent cold exposure. The adipocytes of cold-exposed mice also retain epigenetic memory, demonstrating preparedness for thermogenic gene activation upon subsequent cold exposure [85], consistent with the results of our study.

*AEBP1* is a ubiquitously expressed multifunctional gene that regulates adipogenesis, inflammation, macrophage cholesterol homeostasis, mammary gland development, and atherogenesis [87]. *AEBP1* functions in adipocyte differentiation by negatively regulating the adipose P2 (*aP2*) gene, with high expression of *AEBP1* inhibiting adipogenesis [88]. *AEBP1*-null mice are resistant to diet-induced obesity, indicating that *AEBP1* plays a key role in regulating body fat [89]. In this study, we found that the intronic CG site of the marker gene *AEBP1* in cluster 1 was hypomethylated (Supplementary Tables S8 and S9, Supplementary Figure S1B). These findings suggest that on day 10 after cold exposure and the return to ambient temperature, white adipocytes were more likely to accumulate in the experimental group than in the control group.

The chromatin accessibility results indicate that in pigs recovering from cold exposure by the return to normal temperatures, the differentially expressed genes were concentrated in the TSS region (Figure 6E), a factor that can facilitate a rapid response to subsequent cold exposure. Additionally, in the experimental group, differential peaks were identified within 3 kb of the *UCP2*/*3* and *AEBP1* gene promoters (Figure 6D). The above findings demonstrate that the identified cells exhibit characteristics of brown adipocytes, as indicated by their expression of the UCP2/3 genes.

### 3.5. The Distinctions and Connections Between Beige and Brown Adipocytes

The adipocytes identified in this study were brown, not beige, adipocytes for the following reasons. First, adipose tissue primarily comprises two main cell types: white and brown adipocytes [1,3,4,19,90]. White adipocytes represent the majority, and beige adipocytes are relatively rare [62]. The results of this study indicate that, compared to those in the control group, the number of connective tissue cells in the cold exposure group was significantly increased, whereas the number of adipocytes was significantly decreased by 17.74% (Figure 2F,G). This substantial change in cell number can only be attributed to brown adipocytes, not beige adipocytes.

Second, there is still no consensus on how beige and brown adipocytes are derived from white adipocytes. One view is that under cold exposure, white adipocytes first convert into brown adipocytes to produce a large amount of heat, and then beige adipocytes arise to produce a relatively small amount of heat to maintain body temperature via the precise action of the ubiquitin ligase *RNF20* [91]. However, most researchers believe that beige adipocytes are intermediates in the conversion of white adipocytes to brown adipocytes [24,52,61,92]. In this process, white adipocytes are thought to first be converted to unstable beige adipocytes, and the activation of beige adipocyte browning by MYPT1-PP1β leads to their conversion into brown adipocytes [93]. Moreover, the conversion of white adipocytes to both brown and beige adipocytes is regulated by a common set of genes, including *GRAF1* [1], *RNF20* [91], *TRIM56* [16], and *FBXW7* [94]. Regardless of how beige and brown adipocytes are interconverted, this study demonstrated that cold exposure induced a reduction in the number of adipocytes (Figure 2G). This reduction comprised the disappearance of white adipocytes after they consumed intracellular lipids and were converted into other types of cells, such as preadipocytes. Since the lipids have been completely consumed, the existence of brown adipocytes can be confirmed, although it is not possible to confirm the presence of beige adipocytes.

Third, the marker genes for beige and brown adipocytes differ. The scRNA-seq analysis in this study did not detect the beige adipocyte marker gene *CD137* [20]. It is generally believed that white adipocytes contain a single large lipid droplet; beige adipocytes have relatively smaller lipid droplets, and brown adipocytes contain very small lipid droplets [1,12,62]. This study successfully obtained evidence of intracellular lipid droplets in mouse brown adipose tissue sections through HE staining (Figure 3B). However, the evidence from HE staining of intracellular lipid droplets in adipocytes in pig adipose tissue was unclear (Figure 2D). Fortunately, transmission electron microscopy confirmed the presence of microlipid droplets in adipocytes in the cold exposure group (Figure 4G). Considering the above results, the identified cells were confirmed as brown adipocytes.

### 3.6. Limitations

After HE staining, the numbers of connective tissue cells and adipocytes were counted separately. It is possible that some brown adipocytes whose lipid droplets had been consumed and thus appeared fibroblast-like in shape were counted as connective tissue cells. Thus, the method has certain limitations. However, in terms of the overall trend, the error rate did not exceed 5%, i.e., within the 95% confidence interval [95].

Visible connective tissue was not removed from the sample used for scRNA-seq analysis. We previously conducted an experiment in which we carefully removed visibly apparent connective tissue during qRT-PCR, as it could indicate UCP2/3 (gene and protein) expression, and the levels in the cold exposure group were significantly higher than in the control group. Thus, although the volume of connective tissue was low, the cell count was high, creating considerable background noise. However, to ensure that other laboratories can replicate the results of this study, we have maintained the current analytical procedures.

During the experimental process, neither water nor food intake was restricted. Cold stimulation led to an increase in food intake, and the cold exposure group consumed more food, which helped maintain body temperature. However, since we achieved our experimental goal of identifying adipocytes, we did not consider conducting an additional experiment with restricted feeding. Moreover, we deemed restricted feeding inappropriate from an animal welfare perspective.

## 4. Materials and Methods

### 4.1. Ethics Statement

The swine and mouse research was approved by the Committee for Animal Welfare of the Institute of Animal Husbandry, Heilongjiang Academy of Agricultural Sciences (HAAS) (No. NKYXMS-20240517, 17 May 2024, NKYXMS-20240912, 12 September 2024), in accordance with the Laboratory Animal Guideline for Ethical Review of Animal Welfare (GB/T 35892-2018). The experimental procedures were designed to minimize animal pain and stress to the greatest extent possible, ensuring animal welfare.

### 4.2. Human Database Search Strategy and scRNA-Seq Data Analysis

The GEO database (www.ncbi.nlm.nih.gov) was searched for single-cell sequencing results of human white and brown adipose tissue. Data from six adipose tissue samples (GSE227635; white adipose tissue (GSM7104412-4) and brown adipose tissue (GSM7104415-7)) were downloaded [3]. The data from the six adipose tissue samples were merged into a single dataset, and then the R packages (version 4.4.1) “Seurat”, “SingleR”, “limma”, and “GSVA” were used to conduct a t-SNE clustering analysis on the obtained dataset [3]. The log_2_FC value was set at 1, and the adjusted *p*-value was set at 0.05, with the number of cells with gene expression counts between 200 and 10,000 extracted. Cell type identification was performed with reference to the Human Primary Cell Atlas Data (www.humancellatlas.org), and cluster identification was performed on the basis of adipocyte-specific expressed genes as described previously [4,7]. Considering the high mitochondrial content of brown adipocytes, the percentage of MT RNA was confirmed during the clustering process. Clusters related to cluster 1 were identified based on the maximum log_2_FC values of marker genes of clusters. The percentage of white adipocytes in white and brown adipose tissue was then calculated from the distribution of the six samples in the dataset.

### 4.3. Animals

The experimental swine were raised at the Institute of Animal Husbandry (HAAS) experimental farm, and the cold exposure experiment utilized the controlled temperature and humidity artificial climate chamber of the HAAS. In the first experiment (3 July 2024), 12 Large White pigs were randomly divided into two groups, with individual details listed in Supplementary Table S1. Six pigs were exposed to cold at 10 °C, and six were housed at 25 °C as the control group [27,28,29,30,36]. The pigs were allowed free movement within the artificial climate chamber and had ad libitum access to food and water. The feed formula is described in Supplementary Table S2. The body surface and inguinal temperatures were measured via a smartphone IR thermography device (FLIR One^®^) for 63 h. The pigs were subsequently slaughtered in accordance with animal welfare guidelines, and blood, liver, spleen, longissimus dorsi muscle, groin adipose, and subcutaneous adipose tissues were collected for subsequent experiments (Supplementary Table S1).

Owing to the unexpected discovery of brown adipocytes in the pigs, it was necessary to compare and confirm these cells with those in the classic brown adipose tissue of mice. In a second experiment, 12 Kunming mice were randomly divided into two groups, with individual details listed in Supplementary Table S3. Six mice were placed in cages in the artificial climate chamber and exposed to cold at 8 °C, whereas the other six were housed at 25 °C as the control group [7]. The mice were allowed free movement and had ad libitum access to food and water. After four days, the mice were sacrificed in accordance with animal welfare guidelines, and interscapular adipose tissue was collected for hematoxylin—eosin (HE) staining (Supplementary Table S3).

Imaging at the cellular subunit level was necessary to confirm that cold exposure induced browning of swine adipocytes. To confirm the changes in the subcellular structure of brown adipocytes and the impact of epigenetic modifications on the browning process of porcine adipocytes, a third experiment (25 November 2024) was conducted with 20 Large White pigs randomly divided into two groups, replicating the aforementioned experiment. At the 63rd hour, five pigs from each group were randomly selected and slaughtered in accordance with animal welfare guidelines. The groin adipose tissue from three pigs (two from the cold exposure group and one from the control group) was used for transmission electron microscopy and scanning electron microscopy analyses. The remaining 10 pigs (five from the cold exposure group and five from the control group) were housed at 25 °C for up to 10 days after the termination of cold exposure. The pigs were then slaughtered in accordance with animal welfare guidelines, and groin adipose tissue from six pigs (three from the cold exposure group and three from the control group) was used for ATAC–seq and whole-genome bisulfite sequencing (DNA methylation) (Supplementary Table S4).

### 4.4. scRNA-Seq Data Processing of Swine Inguinal Fat

Single-cell RNA from six samples was isolated via the TaiM 4 droplet generator (https://c.solargenomics.com, ANNOROAD, Beijing, China). The droplets were generated with mRNA capture magnetic beads, and droplet identification microbeads were used for recognition. A DNBSEQ C library was constructed, comprising a cDNA library and an oligo library derived from the mRNA capture magnetic beads and the droplet identification microbeads. Sequencing was performed on the DNBSEQ T7 platform using a PE150 sequencing strategy. The subsequent data analysis methods were consistent with those used for human scRNA-seq analysis.

### 4.5. Adipocyte Size and Count Analysis

The inguinal fat, liver, spleen, and longissimus dorsi muscle from six pigs were fixed, sectioned, and stained with HE. Images were taken with an Axioplan 2 microscope (Carl Zeiss). The images of inguinal fat were assessed with QuPath (version 0.5.1) [95]. Specifically, two tissue components were detected in the sections: (1) the connective tissue area, primarily comprising areas with dense cell nuclei, and (2) the adipocyte area, demonstrating many lipid droplets. The proportions of adipocytes and connective tissue cells in the cold exposure and control groups were statistically analyzed according to the counting results.

### 4.6. Transmission and Scanning Electron Microscopy

In the third experiment, inguinal fat from three pigs in the cold exposure group (*n* = 10) and three in the control group (*n* = 10) was collected and fixed in 2% (*v*/*v*) glutaraldehyde in 100 mM phosphate buffer. The samples were subsequently used for transmission and scanning electron microscopy, with a focus on identifying brown adipocytes at the junction of adipose and connective tissues based on the results of previous experiments. Scanning electron microscopy was performed with a Hitachi Regulus 8100, and transmission electron microscopy was conducted with a Hitachi H-7500.

### 4.7. qRT–PCR Analysis of UCP2/3 Expression in Adipose Tissue

Total RNA was extracted from swine inguinal fat using the TRIzol reagent (Invitrogen, 15596026CN, Invitrogen, Waltham, MA, USA). The RNA was used to obtain cDNA using a cDNA synthesis kit (Invitrogen, 11754250). Real-time quantitative PCR (qRT–PCR) was conducted with SYBR™ Green (PowerUp, A25742, Thermo Fisher Scientific, Waltham, MA, USA). The primers used in this experiment are listed in Supplementary Table S5.

### 4.8. Enzyme-Linked Immunosorbent Assay (ELISA)

The collected blood samples were assessed via ELISA (www.boxbio.cn). ELISA was applied to assess triiodothyronine (T3), thyroxine (T4), insulin, glutathione peroxidase, malonaldehyde, total antioxidant capacity, superoxide dismutase (SOD), creatine kinase, cortisol, and free fatty acids (FFAs). The detection methods were conducted according to the instructions of the specific kits (Supplementary Table S6).

### 4.9. Whole-Transcriptome Sequencing to Construct a ceRNA Network

In the first experiment, six pigs were divided into cold-exposure and control groups. The liver, longissimus dorsi muscle, and subcutaneous fat were sampled separately, resulting in a total of 18 samples for whole-transcriptome sequencing. The expression levels of mRNAs, lncRNAs, circRNAs, and miRNAs were determined for each sample via BMKCloud (www.biocloud.net). A ceRNA network was constructed according to the coexpression relationships among mRNAs, lncRNAs, circRNAs, and miRNAs. Specifically, Pearson correlation analysis was used to construct coexpression networks of mRNAs–lncRNAs, miRNA–circRNAs, circRNA–mRNAs, and circRNA–lncRNAs. The screening criteria were an absolute value of the correlation between coexpressed RNA pairs greater than 0.9 and a *p*-value below 0.01.

The method for determining lncRNA target genes involved the use of a Perl script to identify adjacent genes within a 100 kb range upstream and downstream of the lncRNA as cis-target genes. The correlation between lncRNAs and mRNAs across samples was analyzed using Pearson correlation. Genes with an absolute correlation value greater than 0.9 and a p-value less than 0.01 were considered trans-target genes of the lncRNA. The miRNA target genes were annotated via BLAST+ 2.17.0 software to compare the predicted target gene sequences with the NR, Swiss-Prot, GO, COG, KEGG, KOG, and Pfam databases to obtain the annotation information of the target genes. Subsequently, RNAhybrid (https://bibiserv.cebitec.uni-bielefeld.de/rnahybrid accessed on 28 September 2025) was employed to simulate docking. Given that circRNA molecules have multiple miRNA binding sites, the circRNA host gene was inferred by using TargetScan software (https://www.targetscan.org/vert_80/, accessed on 20 August 2025) to collect miRNAs associated with the circRNA.

### 4.10. DNA Methylation

Whole-genome bisulfite sequencing (WGBS) was conducted according to standard methods [96,97]. In brief, genomic DNA was extracted and subjected to bisulfite treatment. Paired-end sequencing of the samples was conducted on the Illumina platform (Illumina, San Diego, CA, USA). Bismark software (version 0.24.0) was used to align the reads to a reference genome. Differentially methylated regions (DMRs) were identified via DSS software (version 2.12.0). KEGG enrichment analysis of DMR-related genes was conducted to categorize them according to hyper- and hypomethylation status. Further investigation was conducted on the DMRs in the promoters and gene bodies at the CG, CHG, and CHH sites. An integrated analysis of the intersection genes of the scRNA-seq marker genes of clusters in swine was also performed.

### 4.11. ATAC–Seq

ATAC–seq was performed according to a previously reported method [4]. In brief, nuclei were extracted from pig inguinal fat samples and resuspended in a Tn5 transposase reaction mixture. The sequencing of the obtained fragments was conducted on the Illumina platform (Illumina, USA). DeepTools software (version 2.0.1) was used to count ATAC–Seq reads in the gene body and the regions 3 kb upstream and downstream of the transcription start site (TSS). The alignment results were visualized via the Integrative Genomics Viewer (IGV, version 2.19.4). MACS2 (version 2.2.7.1) was employed for peak calling, with a cutoff of *q* < 0.05 for detection. Bedtools (version 2.31.1) was used to merge peaks from different groups, and then the RPM values of samples within each group were calculated. Peaks with |log2fc| > 1 (RPM in the experimental group/RPM in the control group) were considered differentially expressed. The ChIPseeker R package (version 4.4.2) was used to identify genes associated with the differentially expressed peaks.

### 4.12. Molecular Docking of UCP2/3

Molecular docking simulations based on the sequencing results were performed for UCP2/3 with ATP. SWISS-MODEL data (https://swissmodel.ExPASy.org/ accessed on 17 September 2024) were used to convert the sequenced amino acids into protein tertiary structures [27]. The ATP ligand structures were obtained from PubChem. The docking analysis was conducted using AutoDock Vina software version 1.5.6 (https://vina.scripps.edu, accessed on 17 September 2024).

## 5. Conclusions

The goal of this study was to confirm that the adipocytes identified in cold-exposed pigs were brown adipocytes. If it is subsequently demonstrated that these cells are not brown adipocytes, we have instead discovered an adipocyte subclass of a substantial size that is of considerable research importance. The present study first obtained evidence of the existence of swine brown adipocytes via scRNA-seq data analysis. Then, brown adipocytes with an island-like distribution were identified in the HE-stained histological sections of the inguinal fat of cold-exposed pigs. Finally, both scanning and transmission electron microscopy supported the presence of brown adipocytes near the connective tissue in the inguinal fat. Thermogenesis-related *UCP2*/*3* genes were not significantly upregulated in the cold exposure group, and we have discussed the reasons for this phenomenon. In the section concerning limitations, we have explained how to repeat the experiment to significantly increase the protein content of UCP2/3. This study fills a knowledge gap in the research on swine brown fat and can serve as a reference for future studies on fat metabolism.

## Data Availability

All relevant data are contained within the manuscript.

## Supplementary Materials

The following supporting information can be downloaded at: https://www.mdpi.com/article/10.3390/ijms26209871/s1.

## Abbreviations

The following abbreviations are used in this manuscript:

AEBP1	Adipocyte enhancer-binding protein 1
ap2	Adipose P2
ATAC–Seq	Assay for transposase-accessible chromatin high-throughput sequencing
circrna	Circular RNAs
cerna network	Competitive endogenous RNA network
dmrs	Differentially methylated regions
ffas	Free fatty acids
GO	Gene Ontology
IH	H+ leakage
KEGG	Kyoto Encyclopedia of Genes and Genomes
lncrna	Long non-coding RNAs
qrt–PCR	Real-time quantitative PCR
SOD	Superoxide dismutase
T4	Thyroxine
TSS	Transcription start site
T3	Triiodothyronine
UCP1	Uncoupling protein 1
UCP2/3	Uncoupling protein 2/3
WGBS	Whole-genome bisulfite sequencing
