# Supplementary figures and images for: Cold Exposure Induces Swine Brown Adipocytes to Display an Island-like Distribution with Atypical Characteristics
Source: Int J Mol Sci. 2025 Oct 10;26(20):9871. doi: 10.3390/ijms26209871 (PMC12563708; doi:10.3390/ijms26209871)

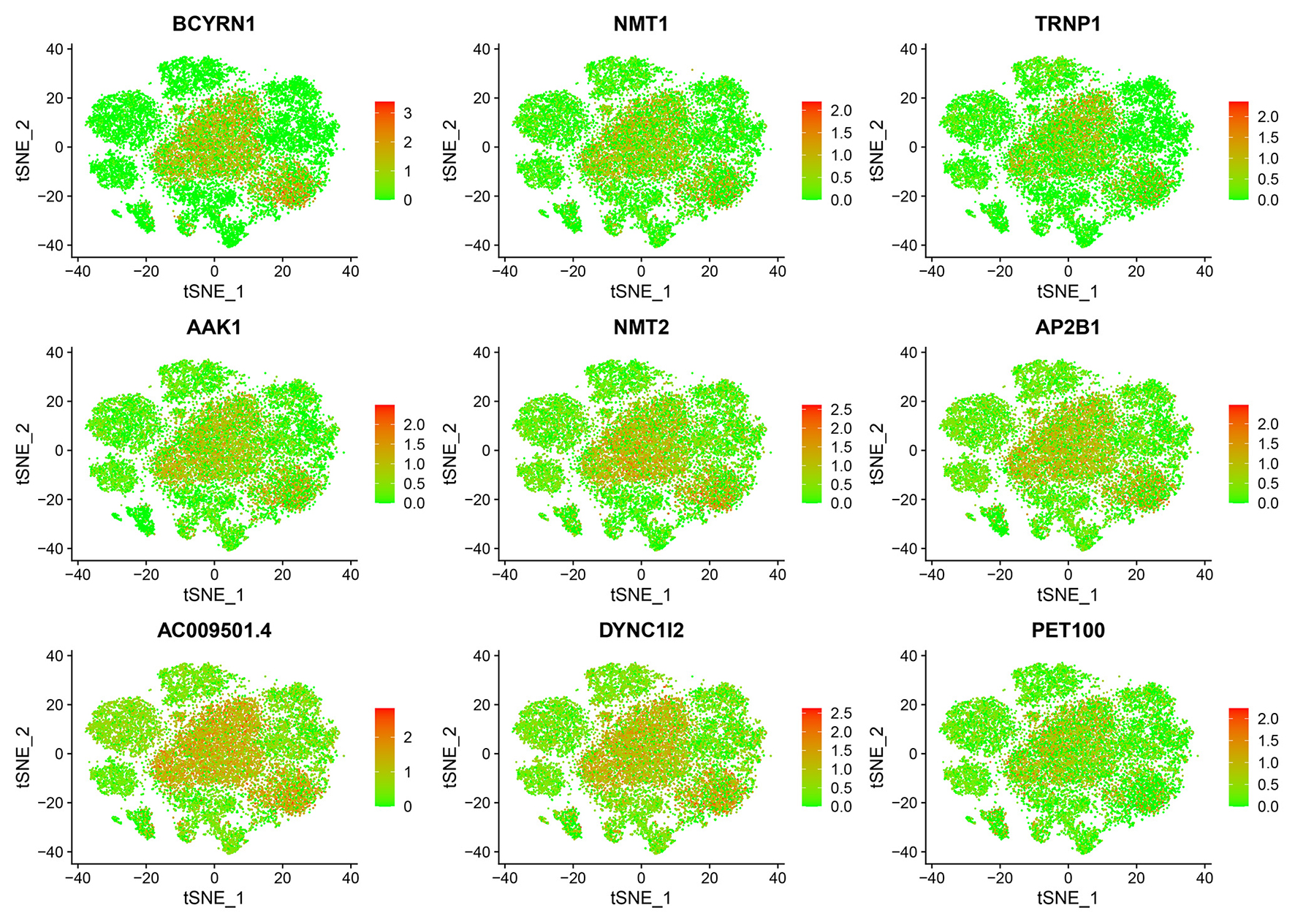

Supplement: Supplementary file 1 [file ijms-26-09871-s001.zip › supplementary fig 1 a human WAT 9 marker genes.jpg]

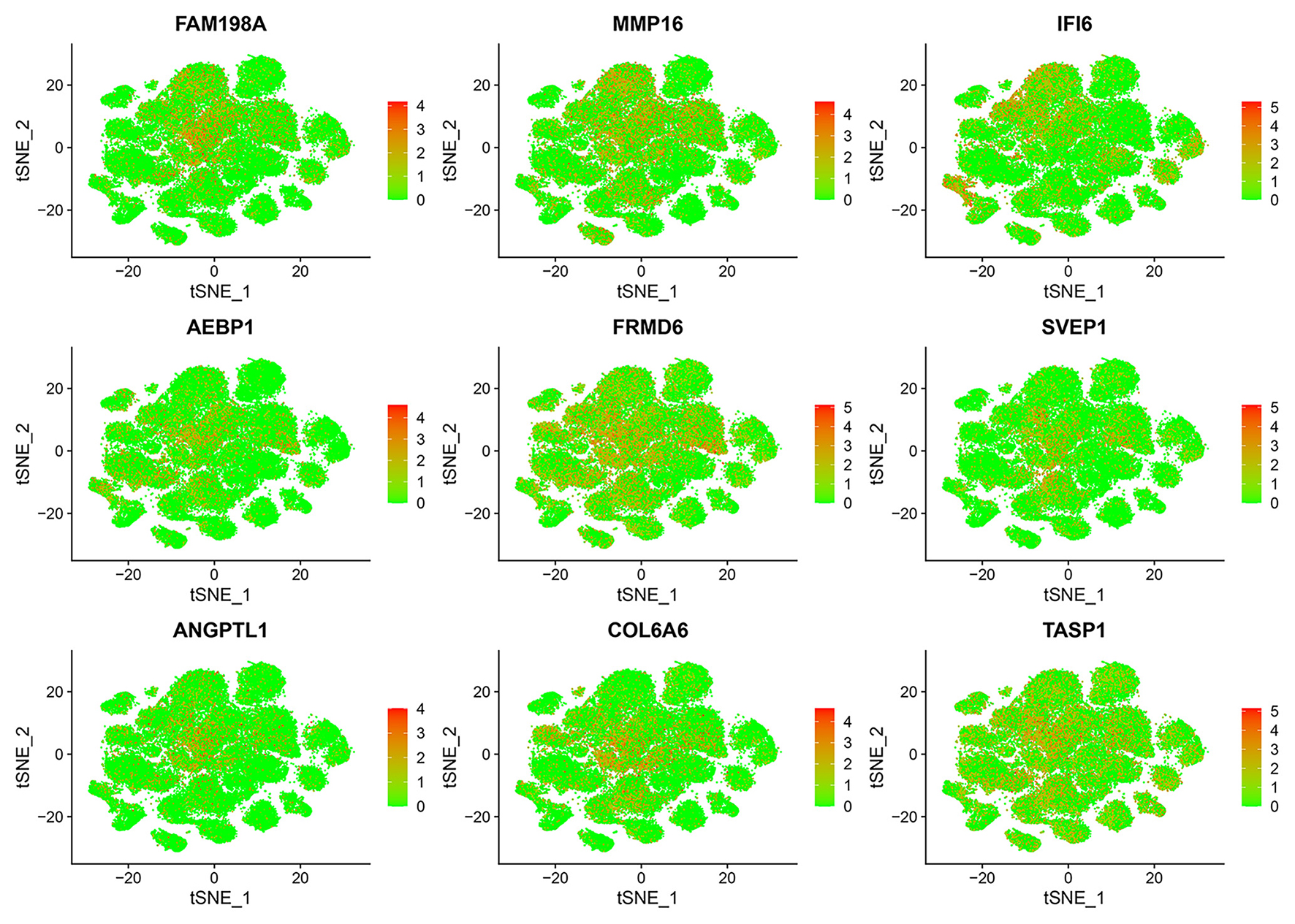

Supplement: Supplementary file 1 [file ijms-26-09871-s001.zip › supplementary fig 1 b swine WAT 9 marker genes.jpg]

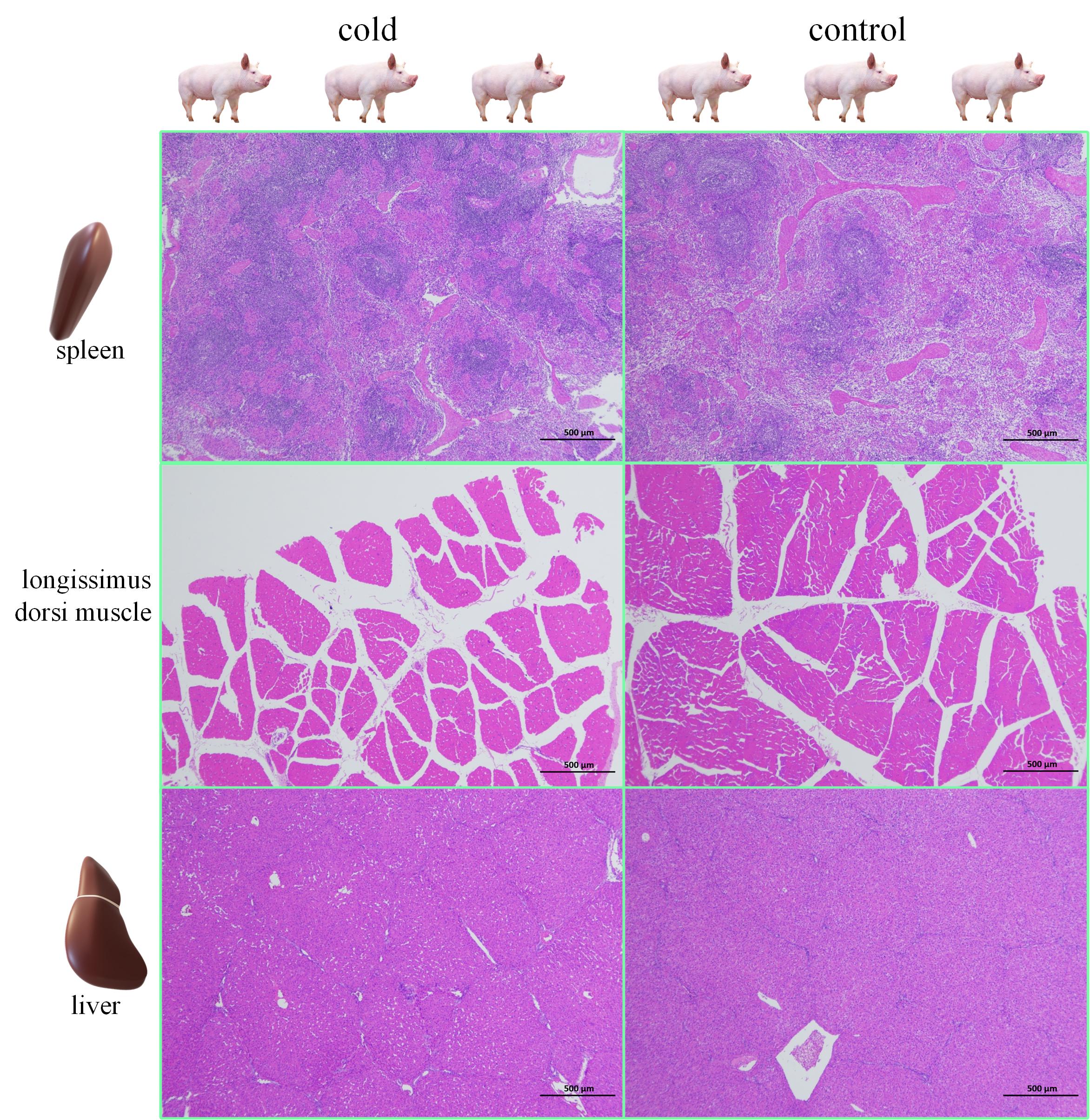

Supplement: Supplementary file 1 [file ijms-26-09871-s001.zip › Supplementary fig 2.jpg]

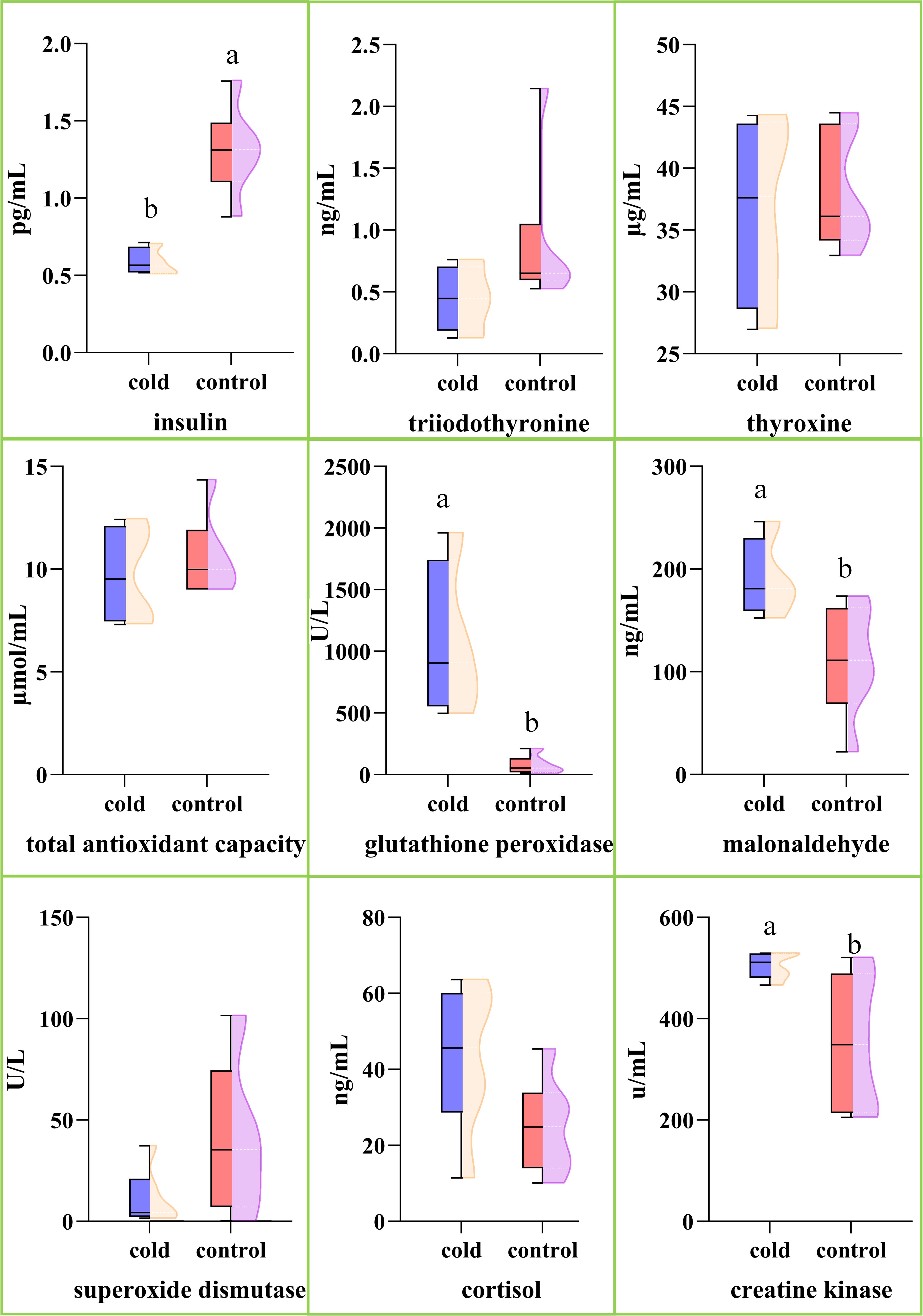

Supplement: Supplementary file 1 [file ijms-26-09871-s001.zip › supplementary fig 3.jpg]

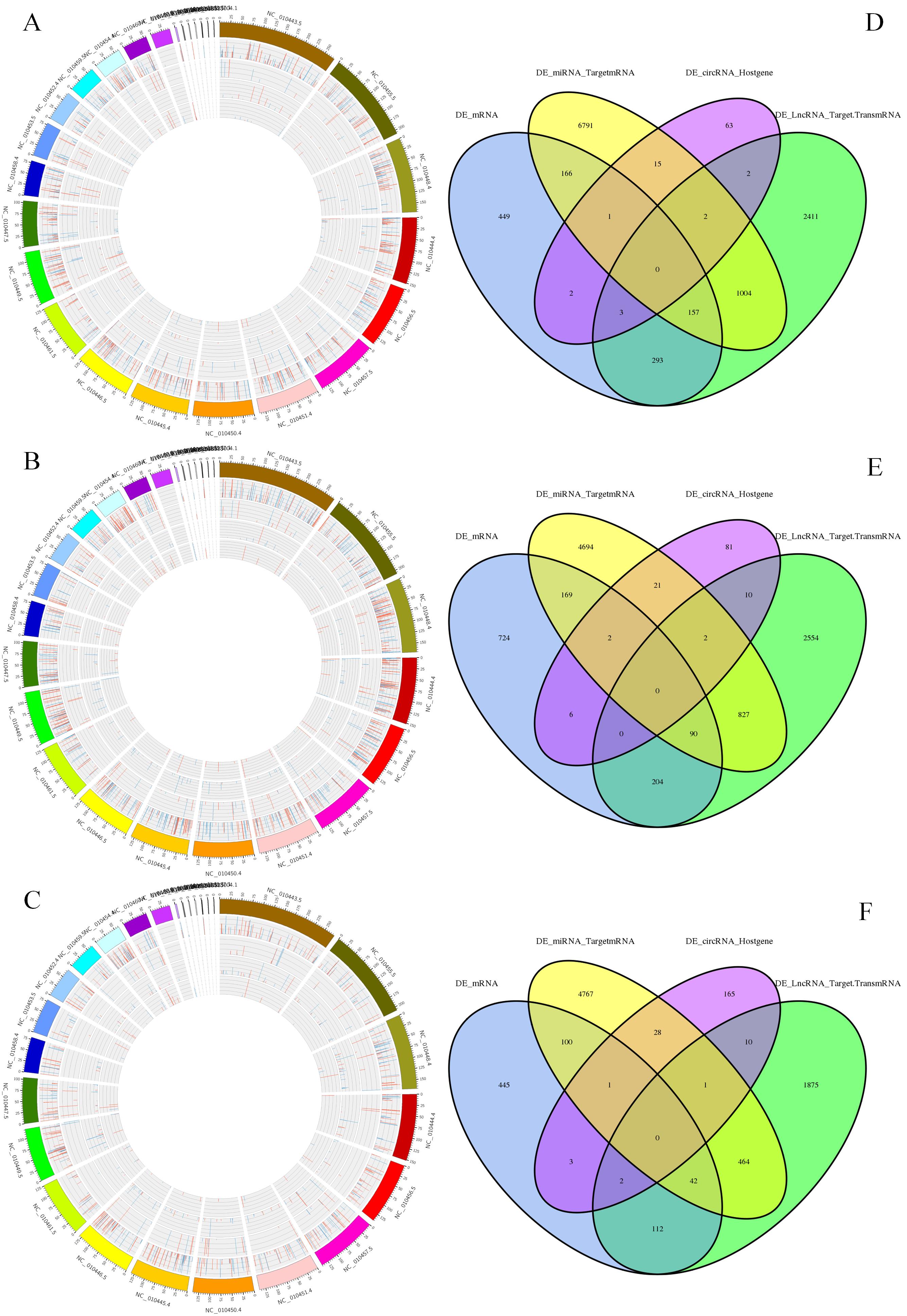

Supplement: Supplementary file 1 [file ijms-26-09871-s001.zip › Supplementary fig 4.jpg]

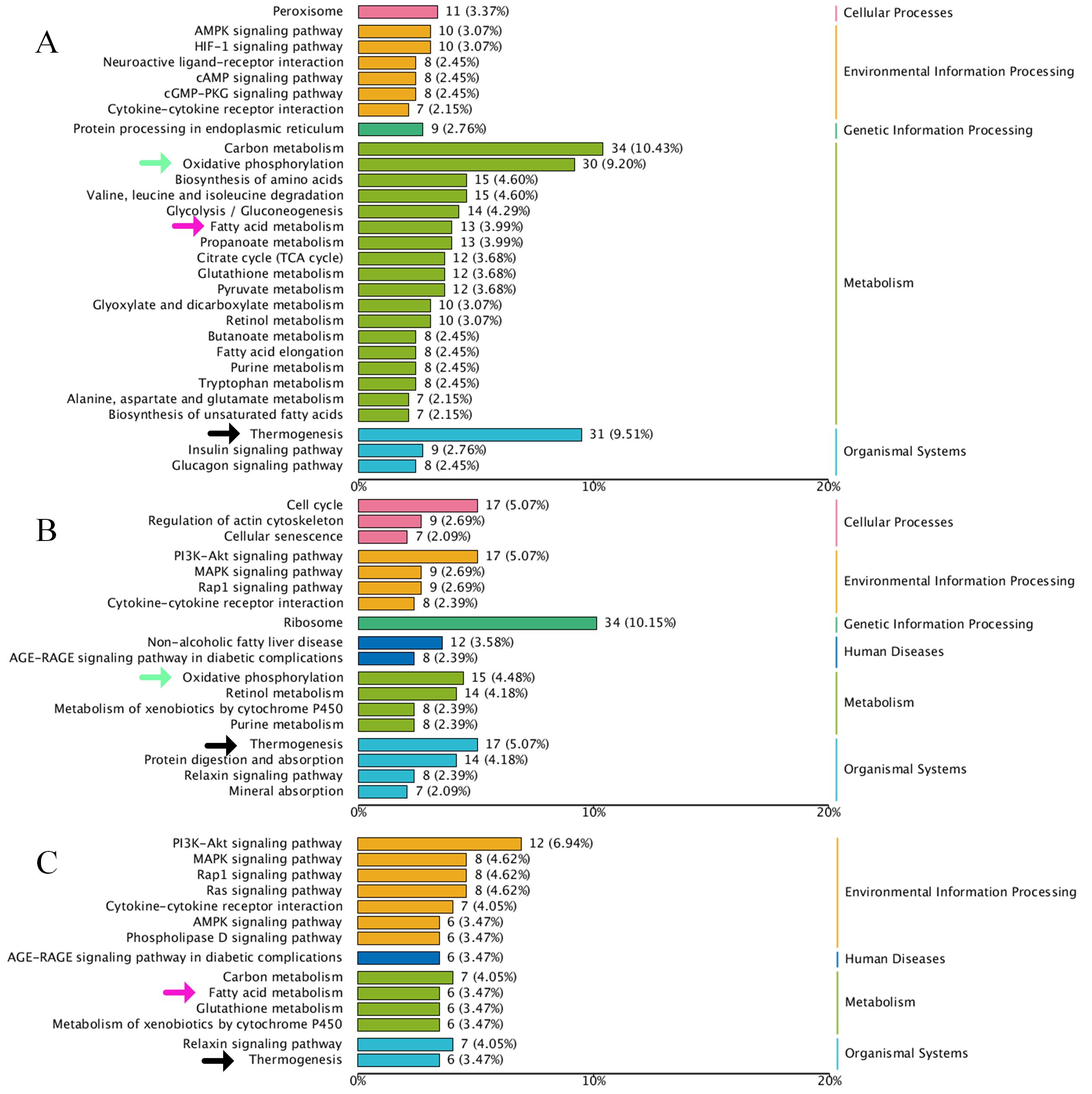

Supplement: Supplementary file 1 [file ijms-26-09871-s001.zip › Supplementary fig 5.jpg]

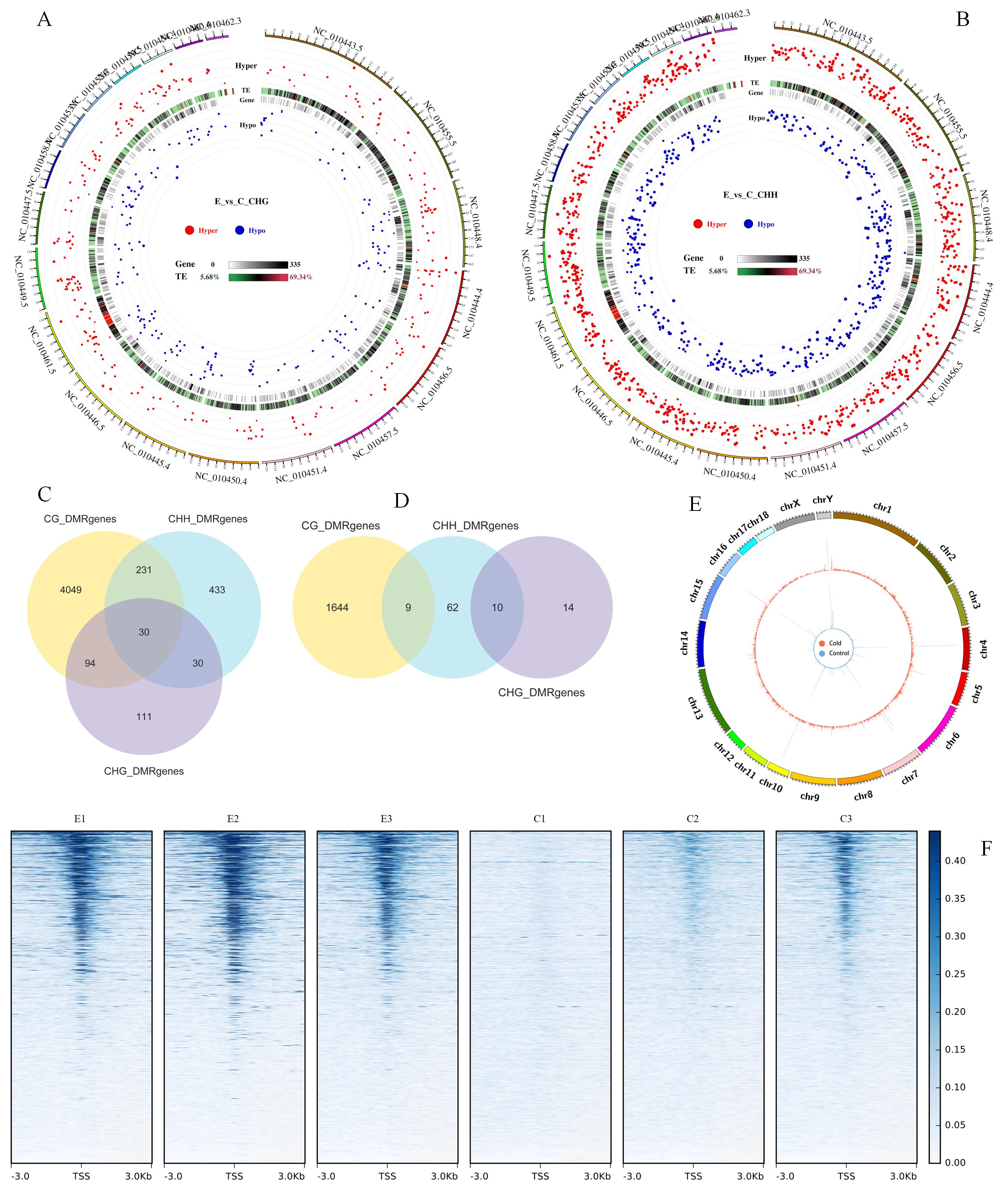

Supplement: Supplementary file 1 [file ijms-26-09871-s001.zip › Supplementary fig 6.jpg]
